# Net greenhouse gas balance with cover crops in semi-arid irrigated cropping systems

**DOI:** 10.1038/s41598-022-16719-w

**Published:** 2022-07-20

**Authors:** Pramod Acharya, Rajan Ghimire, Wooiklee S. Paye, Amy C. Ganguli, Stephen J. DelGrosso

**Affiliations:** 1 Department of Plant and Environmental Science, New Mexico State University, Las Cruces, NM 88003 USA; 2 Agricultural Science Center, New Mexico State University, 2346 State Road 288, Clovis, NM 88101 USA; 3 Department of Animal and Range Sciences, New Mexico State University, Las Cruces, NM 88003 USA; 4 Soil Management and Sugar Beet Research Unit, USDA-ARS, Fort Collins, CO 80526 USA

**Keywords:** Agroecology, Biogeochemistry, Climate-change mitigation

## Abstract

Climate smart agriculture has been emphasized for mitigating anthropogenic greenhouse gas (GHG) emissions, yet the mitigation potential of individual management practices remain largely unexplored in semi-arid cropping systems. This study evaluated the effects of different winter cover crop mixtures on CO_2_ and N_2_O emissions, net GHG balance (GHG_net_), greenhouse gas intensity (GHGI), yield-scaled GHG emissions, and soil properties in irrigated forage corn (*Zea mays* L.) and sorghum (*Sorghum bicolor* L. Moench) rotations. Four cover crop treatments: (1) grasses, brassicas, and legumes mixture (GBL), (2) grasses and brassicas mixture (GB), (3) grasses and legumes mixture (GL), and (4) a no-cover crop (NCC) control, each replicated four times under corn and sorghum phase of the rotations, were tested in the semi-arid Southern Great Plains of USA. Results showed 5–10 times higher soil respiration with cover crop mixtures than NCC during the cover crop phase and no difference during the cash crop phase. The average N_2_O-N emission in NCC was 44% lower than GL and 77% lower than GBL in corn and sorghum rotations. Cash crop yield was 13–30% greater in cover crop treatments than NCC, but treatment effects were not observed for GHG_net_, yield-scaled emissions, and GHGI. Integrating cover crops could be a climate smart strategy for forage production in irrigated semi-arid agroecosystems.

## Introduction

The Paris Climate Agreement in 2015 aimed to limit global warming by holding global average temperature rise below 2 °C by 2100 compared to pre-industrial levels^1^. The agricultural sector contributes to global warming, emitting 10–12% of the total anthropogenic GHG emissions^2,3^. This number could increase to 20–25% if emissions from land-use change are included, and up to 34% if up and downstream products are included^4–6^. In addition, the agriculture sector accounts for approximately 56% of the total anthropogenic non-CO_2_ GHG emissions globally^6,7^. Soil emits about ten times more CO_2_ than burning fossil fuels, but the soil emission is roughly balanced by a similar amount of C fixation from photosynthesis and it also has the largest reservoir of carbon (C) in terrestrial ecosystems^8,9^. Although GHG emissions via natural processes are inevitable, a substantial reduction in anthropogenic GHG emissions is possible through agricultural innovations^10^. About 60% of the global anthropogenic N_2_O emissions come from agriculture, primarily through synthetic fertilizer and manure application^3^. Although agricultural soils are considered a source of N_2_O on annual or greater time scales, some studies suggest soils can be a sink if climate smart agricultural practices are adopted^11–13^. Studies have reported up to 116 g ha^−1^ day^−1^ atmospheric N_2_O uptake in nitrogen-limited conditions^12^.

Cover cropping is considered a climate smart strategy to enhance soil health and mitigate global warming because they photosynthetically capture atmospheric CO_2_-C and ultimately store a portion in soils^14^. A meta-analysis highlighted the potential of cover cropping to reduce agricultural GHG by 8% while increasing SOC sequestration by 0.12 Pg C per year^15^. The GHG mitigation potential of cover crops is centered on C sequestration, reduction in fertilizer use with legume cover crops, change in albedo, and enhancing agroecosystem resiliency through a wide range of ecosystem services benefits^14,16^. Besides, cover crops suppress weeds and insect pests and capture residual inorganic nitrogen (N) to prevent it from leaching^17–19^. They also reduce soil denitrification potential by scavenging residual N after crop harvest and can reduce GHG emissions by decreasing soil temperature through canopy or residue cover^20,21^. However, the moisture saving due to a mulching effect of cover crop residues could favor CO_2_ and N_2_O emissions. This is because more soil water and increased microbial substrate from decomposing cover crop residues could accelerate microbial mineralization processes^22,23^. Over longer periods, cropping systems that add more crop residues can reduce atmospheric GHG concentration by improving SOC sequestration despite higher CO_2_ emissions in the first few years^24^.

Cover crops that contain multiple species with different functional properties provide numerous ecosystem services^25^. Compared to single-species cover crops or agricultural management with no cover crops, diverse cover crops support higher microbial abundance and diversity^26,27^. Legume cover crops can fix atmospheric N and have a low C:N ratio in their biomass, increasing soil inorganic N, and affecting heterotrophic respiration and N_2_O emissions^26^. Legumes can increase soil N storage or N_2_O emissions by denitrifiers^28,29^. Cover crops with a high C:N ratio, such as grasses, can conserve N by causing net N immobilization and inhibiting denitrification^30^. Although cover crop residues immobilize mineral N for a short time, they ultimately increase labile pools of C and N, affecting CO_2_ and N_2_O emissions^23^. The availability of labile C increases oxidation and creates oxygen-limiting microsites in the soil, thus favoring N_2_O emission through microbial denitrification^23,31^. Compared to sole cover crops, mixtures can benefit cropping systems by increasing total biomass input, improving C:N balance, enhancing soil microbial diversity, and ultimately leading to SOC sequestration and stabilization^19^. Cover crop mixtures may have variable effects in C and N dynamics depending on species, quality, and quantity of residue input. For example, Bodner et al.^32^ found higher N_2_O emissions with the sole mustard cover crop than with mixtures. In contrast, Drost et al.^26^, from an incubation study, reported similar CO_2_ and N_2_O emissions between sole cover crops and 3- and 15-species mixtures. Since C inputs strongly regulate SOC loss or stabilization, management strategies should focus on maximizing inputs, balancing the quality and quantity of residue input, and minimizing SOC loss to maximize long-term SOC storage^33^.

While increasing SOC and mitigating GHG through diverse cover cropping serve as a climate smart solution for humid and sub-humid temperate regions, the C and N cycling in water-limited environments has been challenged by low precipitation and high temperature variability^34,35^. Arid and semi-arid areas contribute to 57% (0.04 Pg C year^−1^ out of 0.07 Pg C year^−1^) of the net global terrestrial CO_2_ sink^36^. Because of increasing global warming and rapid soil degradation, agroecosystems in these regions are gradually turning from a sink to a source of GHGs^37,38^. Studies suggest that cover cropping may improve subsequent cash crop production because of increased water use efficiency in semi-arid irrigated conditions^39–41^. However, in such water-limited regions, the effects of cover crops on cash crop yield depend on management practices, inherent soil properties, and soil type^42^. Cover crops can enhance N cycling and increase C-sequestration due to increased biomass production and recycling^43,44^. This cover crop derived increase in C could increase CO_2_ and N_2_O emissions in the first few years^32,45^. However, lower soil temperature under cover crops than under fallow could create a more conducive environment for C and N storage and cycling in the soils^45,46^. Nevertheless, the limited information on GHG emissions and C and N cycling in arid and semi-arid agroecosystems warrant a comprehensive assessment of soil C and N components, GHG emissions, and soil temperature and moisture dynamics^47^. Cover cropping is considered a viable strategy to mitigate climate change. Therefore, in the context of a limited number of studies evaluating GHG emissions and their climate change mitigation potential, a comprehensive assessment of GHG emissions, soil properties, and their relationship with soil temperature and moisture could benefit the entire arid and semi-arid ecosystems that cover nearly 40% of the global land area.

An improved understanding of GHG emission, net GHG balance (GHG_net_), and yield response of cover crop-integrated cropping systems would help design climate smart cropping strategies in arid and semi-arid regions. The first objective of this study was to evaluate the cover crop effects on CO_2_-C and N_2_O-N emissions, GHG_net_, greenhouse gas intensity (GHGI), and yield-scaled CO_2_ and N_2_O emissions in a cover crop integrated forage corn-sorghum rotation under semi-arid irrigated conditions of the southern Great Plains of USA. The second objective of this study was to evaluate the C input from cover crop biomass, estimate cash crop yields, and monitor seasonal changes in soil inorganic N and labile C. The last objective was to understand how changes in soil inorganic N, labile C, soil temperature, and moisture under alternative management affect CO_2_-C and N_2_O-N emissions in semi-arid agroecosystems. We hypothesized that integrating cover crops in the forage cropping system would decrease GHG_net_, yield-scaled emissions, and GHGI of the cropping system while they would increase soil C and N cycling and improve cash crop production.

## Results

### Average daily and cumulative greenhouse gas emissions

**Figure 1:**
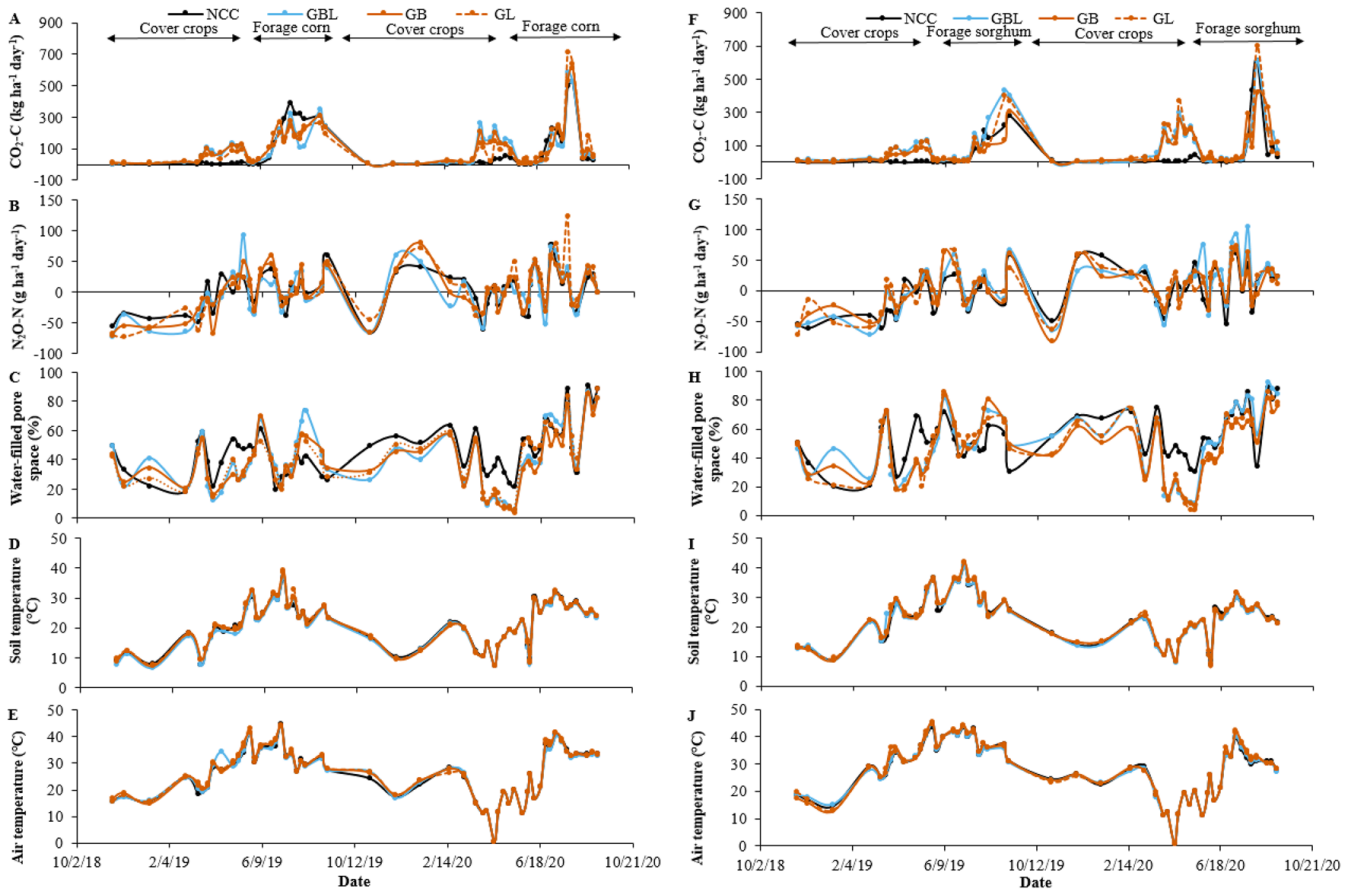
Trend of average CO_2_ and N_2_O emissions, water-filled pore space, soil temperature, and air temperature under cover crop treatments during forage corn and sorghum production from 2018 to 2020. (**A**) Average CO_2_-C emission (kg ha^−1^ day^−1^), (**B**) average N_2_O-N emission (g ha^−1^ day^−1^, (**C**) water-filled pore space (%), (**D**) soil temperature (°C), and (**E**) air temperature (°C) in cover crop-corn rotation, and (**F**) average CO_2_-C emission, (**G**) average N_2_O-N emission, (**H**) water-filled pore space (%), (**I**) soil temperature (°C), and (**J**) air temperature (°C) in cover crop-sorghum rotation. *NCC* no cover crops control, *GBL* grasses, brassicas, and legumes mixture, *GB* grasses and brassicas mixture, *GL* grasses and legumes mixture.

**Figure 2:**
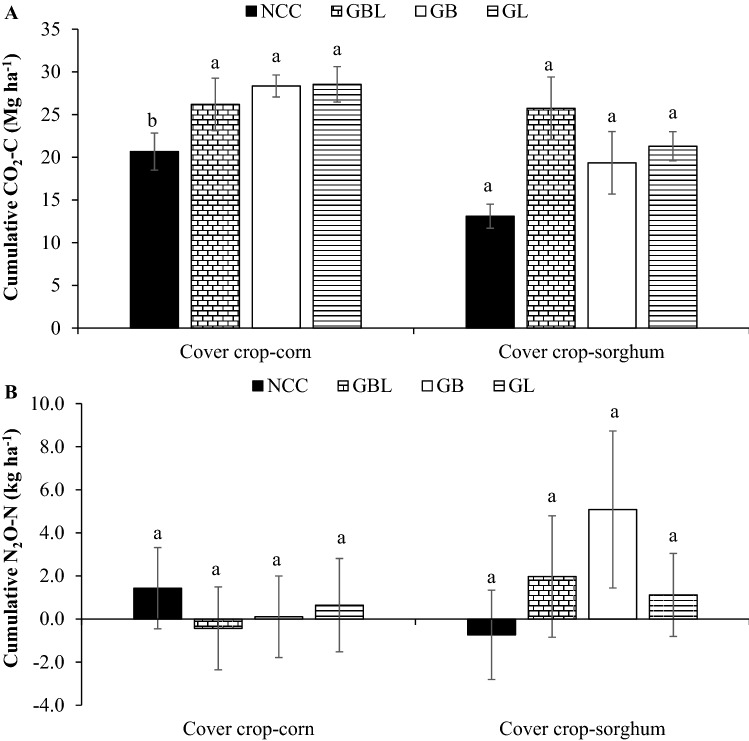
Cumulative CO_2_-C (**A**) and N_2_O-N (**B**) emissions in forage corn and forage sorghum across crop growing phases (cover crops and cash crop) and years (2018/19 and 2019/20). For each rotation, bars with a different letter indicate statistical significance between cover crop rotations at *p* ≤ 0.05 per protected LSD test. Error bars represent the standard error of the mean. *NCC* no cover crops control, *GBL* grasses, brassicas, and legumes mixture, *GB* grasses and brassicas mixture, *GL* grasses and legumes mixture.

Soil CO_2_-C emissions in cover crop-forage corn rotation were consistently higher when the cover crop was present and during the cash crop growing period (Fig. 1A,F). During the cover crop growing period, the difference in CO_2_-C emissions between cover crop treatments and NCC was visible in later growth stages, i.e., from February to April. Soil CO_2_-C emissions, without accounting for SOC inputs from cover crop above- and below-ground biomass addition, in cover crop phases, were 10.3–10.8 times greater in cover crop treatments than the NCC (Table 1). However, CO_2_-C emissions did not vary among treatments during the corn phase of the rotation (Supplementary Table S2). Among phases in cover crop-corn rotation, the greater average fluxes were observed in the corn phase than in the cover crop phase in both years, with 1.6–2.0 times greater emissions in cover crop treatments and 21 times greater emissions in NCC treatment (Table 1). The cumulative CO_2_-C emission under cover crop treatments was 1.27–1.38 times higher than NCC (Fig. 2A). Among years, the average CO_2_-C emission across treatments and sampling dates was 1.98 times higher in 2019/20 than in 2018/19 in the cover crop phase, and no difference between years in the corn phase of the rotation.

**Table 1:** Average CO_2_-C and N_2_O-N emissions under diverse cover crop treatments from 2018 to 2020.

	CO_2_-C emission				N_2_O-N emission			
	Forage corn		Forage sorghum		Forage corn		Forage sorghum	
	Cover crop phase^§^	Cash crop phase	Cover crop phase	Cash crop phase	Cover crop phase	Cash crop phase	Cover crop phase	Cash crop phase
	(CO_2_-C kg ha^−1^ day^−1^)				(N_2_O-N g ha^−1^ day^−1^)			
**Treatment**								
NCC	7.8 ± 2.2b^‡^	162 ± 18.8	10.5 ± 3.4c	85.8 ± 10.6	− 3.4 ± 5.8^‡^	10.2 ± 1.2b	− 13.5 ± 6.4	7.7 ± 3.4b
GBL	84.3 ± 22.7a	136 ± 11.5	80.3 ± 11.9a	113 ± 23.3	− 6.0 ± 4.2	9.2 ± 2.5b	− 13.1 ± 4.3	34.4 ± 12.7a
GB	78.2 ± 8.9a	157 ± 13.1	62.7 ± 10.9b	85.7 ± 26.0	− 8.8 ± 6.3	14.2 ± 1.9ab	− 16.3 ± 23.0	16.1 ± 2.1ab
GL	83.8 ± 16.0a	155 ± 13.1	56.5 ± 5.9b	100 ± 10.8	− 10.2 ± 6.2	18.3 ± 5.2a	− 6.3 ± 5.3	15.3 ± 3.0ab
**Year**								
2018/19	42.6 ± 6.5b	167 ± 8.1	38.1 ± 6.4b	92.6 ± 13.5	− 19.4 ± 2.7b	9.3 ± 1.4b	− 9.1 ± 12.4	10.1 ± 2.0b
2019/20	84.4 ± 15.5a	138 ± 10.6	66.9 ± 9.6a	99.9 ± 13.1	5.3 ± 1.9a	16.6 ± 2. 7a	0.8 ± 2.5	26.7 ± 6.6a

*NCC* no cover crops control, *GBL* grasses, brassicas, and legumes mixture, *GB* grasses and brassicas mixture, *GL* grasses and legumes mixture where grasses include annual ryegrass and triticale, brassicas include daikon radish and turnip, and legumes include berseem clover and winter pea.

^‡^Mean values (± standard error) followed by different lowercase letters in a column indicate significant differences among cover crop treatments or between years (*p* ≤ 0.05, LSD test).

^§^In the 2018/19 cropping year, the cover crop phase represented the greenhouse gas monitored from November 20, 2018, to May 15, 2019, and the cash crop phase from May 16, 2019, to September 5, 2019, whereas in 2019/20 cropping year, cover crop phase represented greenhouse gas monitored from September 6, 2019, to May 14, 2020, and cash crop phase from May 15, 2020, to September 3, 2020.

In the cover crop-forage sorghum rotation, the higher soil CO_2_-C fluxes were observed when the cover crop was actively growing (spring season) and during the cash crop phase (Fig. 1F). The highest fluxes were observed in the sorghum growth phase, irrespective of the crop year. The average soil CO_2_-C emission across years in the cover crop phase was 5.38–7.65 times higher in cover crop treatments than in NCC, while emissions from GBL were higher than GB and GL treatments (Table 1). During the sorghum growth phase, no significant differences were observed among treatments, with CO_2_-C emissions ranging between 85.7 to 113 kg CO_2_-C ha^−1^ day^−1^. Also, the cumulative CO_2_-C emission for the entire cover crop-sorghum rotation period did not differ among treatments (Fig. 2A). Between study years, average soil CO_2_-C emission was 1.76 times higher in 2019/20 than in 2018/19 during the cover crop phase, while no difference was observed during the sorghum phase of the cover crop-sorghum rotation.

Averaged across the study years, soil N_2_O-N emissions were inconsistent in the cover crop and corn phases (Fig. 1B). The emissions were negative during the cover crop phase and positive during the main crop phase, with the values between − 10.2 and − 3.42 g N_2_O-N ha^−1^ day^−1^ in the cover crop phase and 9.18 to 18.3 g ha^−1^ day^−1^ in the corn phase (Table 1). The average N_2_O-N emission during the main crop phase in the GL mixture was significantly greater (79–99%) than NCC and GBL. The cumulative N_2_O-N emissions in the cover crop-corn rotation were not different among treatments and ranged from − 0.43 to 1.43 kg ha^−1^ (Fig. 2B). Between study years, soil N_2_O-N emissions were 127% and 78% greater during cover crop and corn growth phases, respectively, in 2019/20 than the respective phases in 2018/19.

Soil N_2_O-N emissions in the cover crop-sorghum rotation varied during both phases of the crop rotation and years (Fig. 1G). The average emissions were negative during the cover crop phase and positive during the main crop phase. Also, the emissions were mostly negative in 2018/19 compared to 2019/20 during the cover crop growing period. There was a high variation in N_2_O-N emissions among treatments during the sorghum phase, with average emissions 4.48 times higher in GBL mixture than in NCC but similar to GB and GL in the sorghum phase (p = 0.06) (Table 1). The emissions during the sorghum phase were 1.64 times higher in 2019/20 than in 2018/19. The cumulative N_2_O-N emissions during the cover crop-sorghum period did not differ among treatments, and the values ranged between − 0.73 and 5.08 kg N_2_O-N ha^−1^ (Fig. 2B).

### Soil and air temperatures and soil moisture

In the cover crop-corn rotation, WFPS at 0–0.10 m depth in the cover crop phase was greater in NCC than in cover crops, except during their early growth phase in 2018/19, and the response was opposite during the corn phase (Fig. 1C). The trend of average WFPS varied among treatments and with cropping phases in both years. Average WFPS in NCC, GBL, GB, and GL treatments in the cover crop phase was 41.0%, 28.5%, 28.3%, and 29.5%, respectively, whereas they were 50.5%, 52.7%, 48.0%, and 52.4%, respectively in the corn phase of the rotation. The average soil and air temperature trends were similar among all treatments (Fig. 1D,E). Soil temperature measured during the GHG monitoring ranged between 6.8–39.1 °C in 2018/19 and 7.3–32.5 °C in the 2019/20 cropping season. Correspondingly, the average air temperatures measured at the time of GHG monitoring ranged between 14.9–44.7 °C in 2018/19 and 0.6–41.5 °C.

In cover crop-sorghum rotation, WFPS at 0–0.10 m depth in the cover crop phase was higher in NCC than in other treatments, specifically during their later growth period and after termination (April to May), and the result was reversed during the sorghum phase (Fig. 1H) in both years. Average WFPS in NCC, GBL, GB, and GL treatments were 48.9%, 36.1%, 34.3%, and 34.0%, respectively, in the cover crop phase, while it was 59.0%, 63.8%, 58.6%, and 60.6%, respectively, in the sorghum phase. Soil and air temperatures measured during GHG monitoring were similar among treatments (Fig. 1I,J). In 2018/19, the average soil temperature ranged between 8.7 to 42 °C, while in 2019/20, it was between 6.8 and 31.7 °C. Correspondingly, average air temperatures ranged between 12.7 and 45.2 °C in 2018/19 and 0.6 °C to 42.2 °C in 2019/20.

### Soil properties

The effect of cover crops on SOC_min_ in laboratory incubation was similar to CO_2_-C emission in the field. In cover crop-forage corn rotation, SOC_min_ measured at cover crop termination showed significant differences among treatments, with 134–147% greater SOC_min_ in cover crops than in NCC (Table 2, Supplementary Table S3). Conversely, it was comparable among cover crops and NCC at forage corn harvest. Regardless of treatments, SOC_min_ in 2019/20 was 43% greater during cover crop phase than in 2018/19. In cover crop-forage sorghum rotation, SOC_min_ at cover crop termination was 82–125% greater under cover crops than under NCC, while there was no difference among treatments at sorghum harvest.

**Table 2:** Average soil organic C mineralization (SOC_min_) from incubation study and inorganic N (0–0.10 m) under diverse cover crop treatments.

	SOC_min_				Inorganic N			
	Forage corn		Forage sorghum		Forage corn		Forage sorghum	
	Cover crop termination ^§^	Cash crop harvest	Cover crop termination	Cash crop harvest	Cover crop termination	Cash crop harvest	Cover crop termination	Cash crop harvest
	(kg ha^−1^)				(kg ha^−1^)			
**Treatment**								
NCC	36.4 ± 6.3b^‡^	29.9 ± 5.1	44.6 ± 8.6b	26.5 ± 3.0	17.6 ± 1.4a^‡^	4.1 ± 1.1	18.5 ± 2.1a	3.9 ± 0.9
GBL	89.8 ± 7.4a	42.4 ± 6.4	83.4 ± 6.9a	44.8 ± 8.3	9.2 ± 1.6b	3.4 ± 0.8	10.4 ± 2.2b	6.2 ± 2.3
GB	85.5 ± 9.1a	34.0 ± 1.8	100.3 ± 5.5a	48.6 ± 6.9	8.2 ± 1.7b	3.8 ± 0.9	11.6 ± 3.3b	4.6 ± 0.9
GL	85.1 ± 15.4a	34.0 ± 3. 6	81.0 ± 9.6a	39.7 ± 7.3	6.8 ± 0.5b	3.7 ± 0.7	8.0 ± 1.3b	5.2 ± 1.6
**Year**								
2018/19	61.0 ± 6.7b	32.5 ± 4.1	79.0 ± 7.4	45.0 ± 5.4	12.9 ± 1.4a	1.6 ± 0.1b	15.6 ± 1.8a	2.0 ± 0.3b
2019/20	87.4 ± 9.6a	37.7 ± 2.1	75.6 ± 7.5	34.8 ± 4.3	8.0 ± 1.1b	5.9 ± 0.4a	8.6 ± 1.5b	7.9 ± 1.1a

*NCC* no cover crops control, *GBL* grasses, brassicas, and legumes mixture, *GB* grasses and brassicas mixture, *GL* grasses and legumes mixture.

^‡^Mean values (± standard error) followed by different lowercase letters in a column indicate significant differences among cover crop treatments or between years (*p* ≤ 0.05, LSD test).

^§^Cover crop termination represented soil analysis results sampled on April 18, 2019, and May 15, 2020, whereas cash crop harvest represented soil analysis results sampled on September 26, 2019, and September 19, 2020.

Soil inorganic N (NH_4_^+^ + NO_3_^−^) also varied significantly among treatments at cover crop termination (Table 2). It was 91–158% and 59–131% higher in NCC than in the cover crop mixtures in cover crop-corn and cover crop-sorghum rotation, respectively. However, no significant treatment differences were observed at cash crop harvest, irrespective of the crop rotations. Comparing study years, inorganic N content was 1.61 times higher in 2018/19 than in 2019/20 at cover crop termination before corn planting, and it was 3.61 times higher in 2019/20 than in 2018/19 at corn harvest. Averaged across treatments, inorganic N was 45% lower in 2019/20 than in 2018/19 at cover crop termination time before sorghum planting and 291% higher in 2019/20 than in 2018/19 at sorghum harvest.

### Crop yield, greenhouse gas balance, greenhouse gas intensity, and yield-scaled emissions

Corn biomass yield varied only at *p* = 0.079, where NCC treatment yielded 18.6% lower than GL but comparable to GBL and GB treatments (Table 3, Supplementary Table S4). Sorghum biomass yield in NCC was 18.3–23.0% lower than GBL and GB treatments, while it did not differ from GL treatment. Corn yield was 12.6% higher in 2019/20 compared to 2018/19, while there was no difference in sorghum yield between study years. Average aboveground residue C input from cover crop biomass was similar for both rotations and ranged between 2.0 and 2.4 Mg ha^−1^. In the corn system, C input through cover crops was 17.1% higher in 2019/20 than in 2018/19, and treatment × year interaction was also observed (Supplementary Table S4). Therefore, yield-scaled CO_2_-C and N_2_O-N emissions, GHG_net_, and GHGI were not affected by cover crop treatments in both systems. Yield-scaled N_2_O-N emissions under corn and sorghum systems were higher in 2019/20 than in 2018/19 by 205% and 320%, respectively, with average negative emissions in the first year (Table 3). Average yield-scaled N_2_O-N emissions were negative for GBL and NCC treatments in corn and sorghum systems.

**Table 3:** Crop yield, carbon input, net greenhouse gas balance (GHG_net_), yield-scaled CO_2_-C and N_2_O-N emissions, and greenhouse gas intensity (GHGI) in cover crop integrated forage corn and forage sorghum.

	Corn yield^β^	Sorghum yield	Cover crops aboveground C input	Yield-scaled CO_2_-C	Yield-scaled N_2_O-N	GHG_net_	GHGI
	(Mg ha^−1^)		(Mg ha^−1^)	(kg CO_2_-C Mg^−1^)	(kg N_2_O-N Mg^−1^)	(Mg CO_2_ eq. ha^−1^)	(Mg CO_2_ eq. Mg^−1^ yield yr^−1^)
**Cover crop-forage corn**							
Treatment							
NCC	22.9 ± 1.3b^‡^	–	0	0.95 ± 0.15	0.05 ± 0.08	26.0 ± 1.7	1.18 ± 0.14
GBL	26.8 ± 1.4ab	–	2.4 ± 0.1	1.00 ± 0.13	− 0.03 ± 0.07	22.7 ± 3.8	0.87 ± 0.14
GB	25.9 ± 0.9ab	–	2.0 ± 0.1	1.11 ± 0.08	0.00 ± 0.07	26.6 ± 1.6	1.04 ± 0.08
GL	28.1 ± 1.9a	–	2.2 ± 0.1	1.03 ± 0.07	0.01 ± 0.08	26.4 ± 3.1	0.94 ± 0.10
Year							
2018/19	24.4 ± 1.2b	–	2.0 ± 0.1b	1.11 ± 0.07	− 0.18 ± 0.02b	23. 6 ± 1.4	1.02 ± 0.10
2019/20	27.5 ± 0.8a	–	2.4 ± 0.1a	0.93 ± 0.07	0.19 ± 0.02a	27.3 ± 2.2	0.99 ± 0.07
**Cover crop-forage sorghum**							
Treatment							
NCC	–	24.2 ± 1.6b	0	0.57 ± 0.08	− 0.06 ± 0.09	16. 5 ± 1.8	0.69 ± 0.08
GBL	–	29.7 ± 1.5a	2.3 ± 0.1	0.87 ± 0.11	0.09 ± 0.11	23.7 ± 3.7	0.81 ± 0.12
GB	–	31.5 ± 1.4a	2.1 ± 0.1	0.61 ± 0.12	0.16 ± 0.12	18.7 ± 4.4	0.59 ± 0.15
GL	–	28.3 ± 1.4ab	2.2 ± 0.1	0.76 ± 0.06	0.05 ± 0.07	18.6 ± 2.0	0.66 ± 0.08
Year							
2018/19	–	28.0 ± 1.2	2.2 ± 0.1	0.67 ± 0.07	− 0.10 ± 0.07b	16.3 ± 2.4	0.59 ± 0.08
2019/20	–	28.9 ± 1.3	2.3 ± 0.1	0.73 ± 0.07	0.22 ± 0.04a	22.4 ± 1.9	0.79 ± 0.07

*NCC* no cover crops control, *GBL* grasses, brassicas, and legumes mixture, *GB* grasses and brassicas mixture, *GL* grasses and legumes mixture.

^‡^Mean values (± standard error) followed by different lowercase letters in a column indicate significant differences among cover crop treatments and study years (*p* ≤ 0.05, LSD test) of treatments.

^β^The corn and sorghum yield data were adapted from Paye et al.^40,41^, respectively.

### Relationships among greenhouse gas emissions, soil and air temperatures, and soil moisture

**Figure 3:**
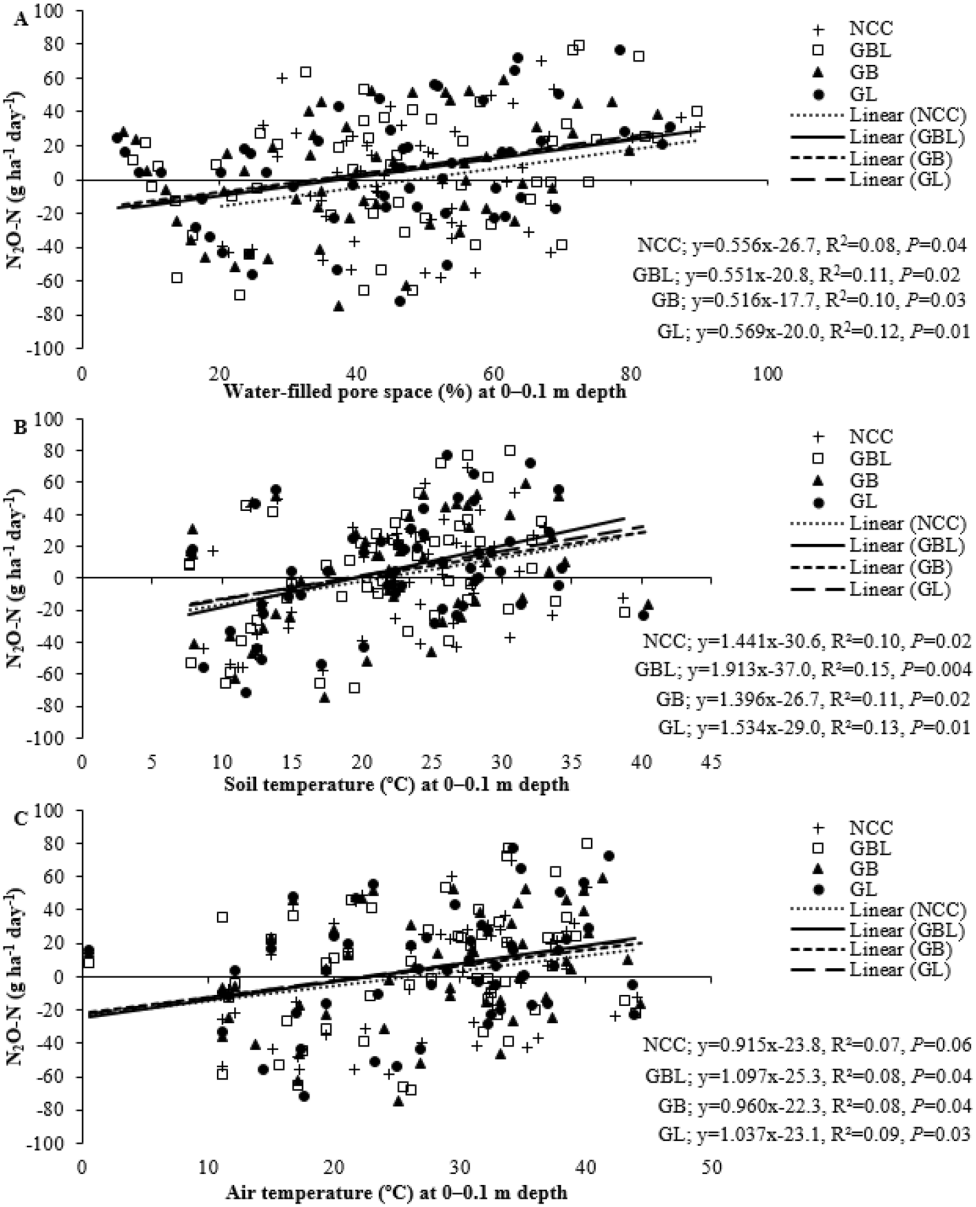
A simple linear regression between N_2_O-N emission and environmental factors: (**A**) with water-filled pore space at 0–0.1 m, (**B**) with soil temperature at 0–0.1 m, and (**C**) with air temperature (n = 212). *NCC* no cover crops control, *GBL* grasses, brassicas, and legumes mixture, *GB* grasses and brassicas mixture, *GL* grasses and legumes mixture.

The N_2_O-N emission had a significant positive relationship with soil temperature, air temperature, and soil WFPS (Fig. 3). The N_2_O-N emission increased by 0.51 to 0.57 g ha^−1^ day^−1^ when WFPS was increased by one percent. Similarly, N_2_O-N emission increased by 1.39–1.91 and 0.91–1.09 g ha^−1^ day^−1^ with a 1 °C rise in soil and air temperature, respectively, in the soil temperature range of 6.8 to 42.0 and air temperature range of 0.6 to 45.2 °C (Fig. 1D,E,I,J). Also, soil N_2_O-N emissions were positively correlated with soil temperature (r = 0.35, p < 0.0001), air temperature (r = 0.28, p < 0.0001), and WFPS (r = 0.31, p < 0.0001) (Supplementary Table S5). In contrast, the regression analysis between CO_2_-C emissions and environmental factors did not show significant relationships (Supplementary Fig. S2). Soil CO_2_-C emissions were positively correlated with soil temperature (r = 0.27, p < 0.0001) and air temperature (r = 0.18, p = 0.011).

## Discussion

Cover crop integration in cropping systems can affect C and N dynamics by improving the diversity and size of the soil microbiome, modifying the soil environment, and reducing the ecological footprint. Evaluation of GHG emissions, C and N inputs from cover crops, the main crop yields, and soil C and N components demonstrated that integrating winter cover crops in irrigated forage corn and sorghum production systems could improve agroecosystem C and N cycling without a significant difference in the net greenhouse gas balance. The average CO_2_ emissions were higher with cover crops than NCC during the cover crop growth period (September to April) due to greater total soil (heterotrophic + root) respiration. Plant root respiration can account for 7–90% of the total soil respiration depending on their growth stage, vegetation type, soil, and climatic conditions^48–50^. However, microbial heterotrophic respiration and soil organic matter decomposition can also be high at the same time due to the rhizodeposition during the growth of the cover crops or the cash crops. It appears biomass recycling after cover crop termination balanced the GHG emissions during the cover crop phase. The CO_2_-C release during the cash crop phases of both rotations and SOC_min_ did not differ among treatments, while cover cropping increased cash crop yields. Higher biomass production and recycling with comparable CO_2_-C fluxes at the system scale suggested the potential to increase SOC storage in the long-term with cover cropping. Aboveground biomass C input of 2.0–2.4 Mg ha^−1^, root-derived C from cover crops, and additional C from cash crop roots can contribute to SOC accumulation in cover cropped rotations.

Integrating cover crops in semi-arid cropping systems also demonstrated the potential to reduce the net N_2_O balance by acting as a sink of atmospheric N_2_O during cover crop growth. The cover crops utilize mineral N for their growth and prevent its loss to the atmosphere as N_2_O emissions. We observed N_2_O uptake in soil, mainly during the cover crop phase of both rotations, and more uptake in cover crop plots than NCC in cover crop-corn rotation. Mostly, the cover crop period of the first year was the sink, while the second year was the source of N_2_O-N emissions. This could be because the second-year cover crop period had residual mineral N from fertilizers applied during first-year cash crops and cover crop decomposition. In contrast, the first-year cover crop did not receive these N credits. The N_2_O uptake occurs when denitrifiers consume N_2_O as an electron acceptor for their respiration or when nitrifiers utilize N_2_O during nitrifier denitrification^12,13^. In N-deficient soils, N_2_O could be the only electron acceptor for complete denitrification leading to N_2_O-N uptake^11^. However, such a phenomenon does not occur when the soil is not N deprived. Nitrogen was not applied in the cover crop phase, but the subsequent cash crops received fertilizer and frequent irrigation. Abundant N in soil and wetting–drying phenomena generally increased N_2_O-N emissions, changing soil from sink to source of nitrous oxide^32^. Legume cover crops can also increase soil N content through N-fixation^29^. Since two of three cover crop mixtures contained legume species, they might have contributed to soil N-accumulation and partly to N_2_O emissions. The soils acted as a source during the cash crop phase when soil moisture was abundant, N was applied, and soil temperature was higher than in the cover cropping phase. A similar N_2_O emissions/uptake response to cover cropping was observed in a silty clay loam under a Mediterranean semi-arid climate that N fertilizer application right after cash crop planting and successive cover crop residue decomposition triggered N loss as nitrous oxide^13^. However, higher N_2_O-N emissions from GL treatment in forage corn and GBL in forage sorghum growing phase than NCC in our study showed a discrepancy in response of various cover crops and highlighted the need for further investigation on factors affecting N_2_O emissions.

The N cycling was improved with cover cropping because N_2_O-N emissions from the NCC treatment were similar to GBL and GB in the corn phase and similar to GB and GL in the sorghum phase of the rotation despite a higher inorganic N under NCC. Nitrogen utilized by cover crops might have been recycled back during cash crop growth and contributed to better nutrient cycling than NCC, leading to higher crop yield in cover crop plots^40,41^. High inorganic N in NCC treatment did not support high forage yield because soil inorganic N content remained similar among treatments at harvest. Forage yield was lower under NCC than cover crop treatments. However, higher inorganic N availability in NCC than cover crop treatments improved forage quality, as shown by higher crude protein content^40^. Cover crops efficiently utilized N from fertilizer input and their residue mineralization than NCC, leading to no difference in net GHG emissions at the system scale.

Both CO_2_-C and N_2_O-N emissions were higher in the second year than in the first year at the system scale. This could be attributed to soil moisture availability, inter-annual climatic variability, and increasing residue input in the second year. The second-year cropping had less precipitation and higher summer temperatures than the first year. Hence, more irrigation was provided in the second year than in the first year resulting in total irrigation + precipitation of 810-mm and 1040-mm in the first and second years, respectively. Studies demonstrated positive linear relationships between soil moisture, temperature, and substrate availability with CO_2_ and N_2_O emissions^28,32,45,51^. In this study, correlation analysis showed that CO_2_-C emissions positively related to soil and air temperature (Supplementary Table S5). In contrast, N_2_O-N emissions varied more with WFPS than with air and soil temperature suggested by both correlation and regression analysis (Fig. 3, Supplementary Table S5). Soil respiration surges with increasing soil temperatures but often declines after the temperature crosses 30 °C^46^. In this study, soil temperature measured at the time of GHG monitoring ranged between 6.8 and 42 °C in different seasons, suggesting a significant role of temperature in CO_2_ emissions in various treatments. Therefore, the average CO_2_ fluxes in this study were higher than studies by Sanz-Cobena et al.^13^ and Mosier et al.^52^, while it was lower than or comparable with others^45,51^.

The N_2_O fluxes in this study had wide variations between crops and years. Soil releases N_2_O gas during both nitrification and denitrification^30,44^. Denitrification potential of soil increases with higher WFPS, usually above 60%, leading to greater N_2_O-N emissions, whereas aerobic nitrification dominates when WFPS falls between 30 and 60%^53^. In this study, WFPS was between 5 and 90%, indicating both nitrification and denitrification processes controlling N_2_O-N emissions. A significant positive correlation of N_2_O-N emission with WFPS, air temperature, and soil temperature also reflected the complex interaction between soil moisture, temperature, and N_2_O-N emissions. These interactions may have influenced CO_2_-C and N_2_O-N emissions differently. Unlike results reported in some studies (e.g., Guardia et al.^30^), we did not observe correlations among CO_2_-C and N_2_O-N emissions, suggesting the need for further research on the role of environmental factors in regulating GHG emissions.

Cover cropping could be a climate smart strategy to improve soil health and increase crop production in arid and semi-arid irrigated cropping systems. In this study, cover crop mixtures and NCC had a similar environmental footprint for producing crops in semi-arid irrigated conditions indicated by similar GHG_net_ and yield-scaled emissions. In contrast, cover crops increased forage yield by 13–30% over NCC. A higher yield in cover crops than NCC could be attributed to better nutrient cycling and water conservation^27,40^. Similar yield-scaled emissions among treatments were due to relatively low cash crop yield in NCC compared to cover crops, suggesting a positive relationship between GHG emissions and crop yield. The variability in N_2_O-N fluxes also affected yield-scaled N_2_O-N emissions. Specifically, yield-scaled N_2_O-N emissions were positive with cover crops and negative with NCC under cover crop-sorghum. Studies suggest that using legume cover crops can increase N_2_O-N emissions^28^. However, the response of GBL in cover crop-corn rotation, another legume integrated treatment in our study, does not support such argument. This could be due to the poor performance of legumes in the 2018/19 cropping year; the aboveground biomass of grasses and legumes in GL treatment had an average ratio of 94:6, while the biomass production of grasses, brassicas, and legumes in the GBL was in a 63:36:1 proportion. The GHGI was higher for NCC in both rotations: 13–35% higher than cover crop mixtures in the cover crop-corn rotation and 5–17% higher than GL and GB mixtures in the cover crop-sorghum rotation. Overall, while the response of different cover crop treatments was variable, this study suggested an increased environmental pressure of growing forage crops without cover crops than with cover crop integration. With the addition of organic C inputs through cover cropping, the SOC_min_ was also increased, potentially improving microbial activity and soil biological health.

It is important to note that C and N loss from bare soils is solely from antecedent organic matter mineralization or fertilizer input. However, the gaseous loss of C and N from cover crop integrated systems could be contributed by above- and below-ground residue C-inputs, root activity, and root exudates^32^. Although the environmental cost of production remained similar between cover crops and NCC, a 13–30% increase in forage production with cover cropping encourages farmers to integrate cover crops into their cropping systems. This study did not measure CH_4_, which may change the net GHG balance. This warrants further studies to evaluate the climate change mitigation potential of cover cropping systems and their viability as a climate smart agricultural tool for semi-arid production systems. In addition, high spatial and temporal variability in the data also suggests the need for more research in semi-arid regions. Soil health and other ecosystem services benefits of cover cropping systems should also be accounted for while considering cover crops to mitigate rapid soil health degradation and fertility loss in arid and semi-arid regions.

## Conclusions

Cover crop inclusion in forage cropping systems significantly increased CO_2_ and N_2_O emissions and cash crop yield while they had no effects on GHG_net_, GHGI, and yield-scaled CO_2_ and N_2_O emissions compared to NCC. Cover cropping did not necessarily reduce GHG emissions in semi-arid irrigated forage production systems. However, the yield benefits from cover crop plots compared to NCC demonstrate its potential as a climate smart strategy for arid and semi-arid agroecosystems. Soil and environmental factors (soil and air temperature and moisture) affected the relative impact of cover crops on CO_2_ and N_2_O emissions. Compared to NCC, cover crops may have utilized residual N to prevent it from being lost in the environment and increased SOC_min_. Adopting such management practices along with no-tillage management could maintain soil health and support forage producers by increasing farm profitability. Considering the environmental footprints and crop yield potential, this study demonstrates the benefits of integrating cover crops in forage crop-fallow systems. However, more research on soil health, GHG emissions, and environmental variables are suggested to warrant the climate change mitigation potential of cover cropping systems in arid and semi-arid regions.

## Materials and methods

### The experimental site and treatments

The study was established on the Olton clay loam soil (fine, mixed, superactive, thermic Aridic Paleustolls)^54^ at New Mexico State University Agricultural Science Center (ASC), Clovis, NM (34° 35′ 59′′ N, 103° 13′ 06′′ W, and elevation 1368 masl). The study area has a hot, dry, semi-arid environment with an annual average maximum and minimum temperatures of 22.6 °C and 6.1 °C, respectively, and average yearly precipitation of 462 mm. The field was fallow for a year before establishing the study plots in September 2018. Baseline soil samples (0–0.1 m) had inorganic N 1.31 mg kg^−1^, potentially mineralizable N 18.9 mg kg^−1^, potentially mineralizable C by aerobic incubation (SOC_min_) 141 mg kg^−1^, soil pH in 1:1 soil water ratio 7.6, SOC 8.29 g kg^−1^, total N 0.93 g kg^−1^, and electrical conductivity 0.43 dS m^−1^.

The study was conducted in a no-tillage corn and sorghum production system with winter fallow in rotation, fallow starting late September to early May of the subsequent year. Both corn and sorghum were present each year, and cover crops were planted to replace the winter fallow. Four cover crop treatments and four replications were arranged in a randomized complete block design. Treatments were cover crop mixtures of grasses, brassicas, and legumes (GBL), grasses and brassicas (GB), grasses and legumes (GL), and a fallow (no cover crop, NCC). Grasses included annual ryegrass (*Lolium multiflorum* Lam.) and winter triticale (*Triticale hexaploid* Lart.), brassicas included turnip (*Brassica rapa* subsp. *rapa*), and daikon radish (*Raphanus sativus* var. *Longipinnatus*), and legumes included pea (*Pisum sativum* subsp. *arvense* L.) and berseem clover (*Trifolium alexandrinum* L.). Individual plot size was 9.1 m × 12.2 m.

Cover crops were planted each year in mid-September using a double-disc drill opener (Model 3P600, Great Plains Manufacturing, Inc., Salina, KS, USA), maintaining 0.15-m row spacing and 0.02-m seeding depth. Seeding rates for cover crops varied among treatments (Supplementary Table S1). They were determined based on individual seed size and germination potential to maintain a comparable plant population for each species combination. All the cover crops were terminated using a mixture of herbicides, as described in Paye et al.^40^, and the residues were left on the ground.

Forage corn (Pioneer P1828AM, 61,776 plants ha^−1^) and sorghum (Mojo Seed OPAL, 123,553 plants ha^−1^) were planted in mid-May, about three weeks after cover crop termination using a John Deere planter (Deere and Company, Moline, IL, USA) adjusted to a row spacing of 0.76-m. Each year the field was fertilized with a single dose of N fertilizers (urea and ammonium nitrate) at 168.1 kg ha^−1^, P (ammonium phosphate) at 42.0 kg ha^−1^, S (ammonium sulfate) at 28.3 kg ha^−1^, and Zn (chelated zinc) at 7.02 L ha^−1^ within 2 days of cash crop planting. Fertilizer rates were based on the recommended dose for irrigated corn and sorghum silage production adjusted with soil test recommendation in the first year, and the same rate was applied for the rest of the study years. Therefore, the N from cover crop residue mineralization was not accounted for in this study. Irrigation was uniformly provided using a center pivot system for all treatments based on the soil moisture content and crop need. Irrigation was provided only to facilitate cover crop planting, germination, and establishment during the cover crop phase. It was adjusted based on precipitation to meet the crop water needs during the corn and sorghum phases of the crop rotation. First-year (2018/19) cover crops and cash crops received 231 and 272 mm of precipitation, respectively, whereas the second year (2019/20) cover crops and cash crops received 294 and 144 mm of precipitation (Supplementary Fig. S1). Therefore, the first-year cover crops and cash crops received 25- and 301-mm of irrigation, respectively, whereas the second-year cover crops and cash crops received 40- and 551-mm irrigation, respectively. In mid-September, cash crops were harvested using a pull-type forage harvester (Model 3960, John Deere, Moline, IL, USA) with an attached wagon, and biomass samples were collected from 4.57 m in length on two rows. All the forage samples were oven-dried at 65 °C until a constant weight to estimate the dry matter yield.

### Greenhouse gas monitoring

CO_2_ and N_2_O emissions were monitored for two years (2018/19 and 2019/20) once a week during the cash crop growth phase (June to September) and once every two to four weeks during the cover crop phase (October to May) with 25 and 28 measurements done in 2018/19 and 2019/20, respectively. Polyvinyl chloride (PVC) rings of 0.1-m diameter and 0.1-m height were installed in each experimental plot about 0.2-m away from the crop row on all the plots to avoid measurement bias among treatments. The PVC rings were inserted in the ground (0.09-m), leaving 0.01-m headspace above the ground for GHG monitoring. The rings were removed during field operation and re-installed at the same spot immediately after completing the fieldwork. CO_2_ fluxes were measured by using Environmental Gas Monitoring System (EGM-5) portable CO_2_ gas analyzer (PP Systems, Amesbury, MA, USA) connected to a soil respiration chamber (area = 78.5 cm^2^, volume = 1171 cm^3^). Aliquots of air entering the CO_2_ analyzer were passed through a MIRA Pico Laser Analyzer (Aeris Technologies, Hayward, CA, USA) to determine N_2_O emissions. Ambient air CO_2_ and N_2_O were also recorded before starting the measurement in the experimental plots, later subtracted from the experimental plot values to calculate the net GHG flux in each plot. The measurements were done from 08:30 to 11:30 h throughout the study to reduce the variability in GHG emissions due to diurnal temperature variation. Any plants inside the PVC rings at the measurement time were hand-clipped and removed to avoid CO_2_ contribution from aboveground plant parts. Gas emissions rates (*R*) were determined using Eq. (1).

1 $$R=\frac{{G}_{n} - {G}_{0}}{{T}_{n}}\times \frac{V}{A},$$

where *R* is the gas emission rate (CO_2_ or N_2_O flux in g m^−2^ h^−1^), *G*_0_ is the gas concentration (CO_2_/N_2_O) at the time of gas chamber installation (*T* = 0), *G*_*n*_ is the gas concentration at time *T*_*n*_ (200 s), *A* is the area of soil exposed in m^2^, and *V* is the system volume in m^3^. The cumulative emission of CO_2_-C and N_2_O-N was estimated by linear interpolation of weekly/bi-weekly emission rates and numerical integration of individual data points. Hydra-probe SDI-12 (Stevens Water Monitoring Systems, Inc., Portland, OR, USA) was used to estimate air and soil temperatures and moisture content at the surface (0–0.10 m).

### Soil sampling and laboratory analyses

Soil samples were collected from 0 to 0.10 m depth of each plot using a core sampler at the time of cover crop termination (mid-April) and cash crop harvest (mid-September) each year. Four cores were collected from each plot, mixed well, and composite subsamples of ~ 300-g were brought to the laboratory for estimating 72-h SOC mineralization (SOC_min_) by aerobic incubation^55^ and soil inorganic N by ammonia analysis method in a Timberline Instruments, Boulder, CO, USA. Three cores of diameter 0.023-m and 0–0.10 m in depth were collected and oven-dried for 24 h at 105 °C to determine the dry bulk density and gravimetric water content. Inorganic N and SOC_min_ concentrations were converted to a volume-area-based unit using the bulk density values. The water-filled pore space (WFPS) was calculated using Eq. (2):

2 $${\text{WFPS~}}\left( {{\% }} \right) = \frac{{{\uptheta }}_{{\text{g}}} \times {{~\uprho }}_{{\text{b}}} }{{\left( {1 - \frac{{{{\uprho }}_{{\text{b}}} }}{{{{\uprho }}_{{\text{s}}} }}} \right)}} ,$$

where, θ_g_ = gravimetric soil moisture (%), ρ_b_ = bulk density (Mg m^−3^), and ρ_s_ = particle density of 2.65 Mg m^−3^.

### Net greenhouse gas balance, greenhouse gas intensity, and yield-scaled emission

The GHG_net_ from CO_2_ and N_2_O was calculated by using Eq. (3)^56^ below:

3 $${\text{GHG}}_{net} \left(\text{Mg }{\text{CO}}_{2}\text{ eq}.{\text{ ha}}^{-1} {\text{year}}^{-1}\right)={\text{CO}}_{2}\text{ eq}.\text{ of }\left(\text{farm operations }+\text{ farm inputs }+\text{ soil heterotrophic respiration }+ {\text{N}}_{2}\text{O emission}-\text{ crop residue returned to the soil}\right),$$

where CO_2_ eq. of farm operations included installation and use of the center pivot; farm inputs included production, transportation, storage, transfer, and application of fertilizers, pesticides, and herbicides; cash crop and cover crop planting and cash crop harvesting. The CO_2_ eq. of farm operations and farm inputs were calculated using literature values from Lal^57^. Similarly, the heterotrophic respiration was calculated by multiplying measured soil respiration by 0.307 value^48^. The methane emissions were not estimated in this study. Agricultural soils in arid and semi-arid regions are often low emitters or serve as a small sink for CH_4_^52,58^. The CO_2_ equivalent of N_2_O emissions (310) was estimated based on Smith^7^ on a 100-year timescale.

Cover crop biomass yield was estimated by hand-clipping biomass samples from four 0.25-m^2^ areas (total 1-m^2^) per experimental plot and oven drying at 65 °C for 72 h. Approximately 100-g subsamples were ground in a Thomas Wiley laboratory mill (Arthur H. Thomas Company, Swedesboro, NJ, USA) to pass through a 1-mm screen. The ground samples were analyzed for C content in a CN Analyzer (LECO Corporation, St. Joseph, MI, USA) using the dry combustion procedure. CO_2_ eq. of residues returned from cover crops was calculated using biomass C estimate for each treatment. Since cash crops were harvested without leaving residues, we did not account for the C returned from the cash crops. GHG_net_ was divided by the total annual forage yield (Mg ha^−1^) to calculate GHGI. Also, yield-scaled CO_2_-C and N_2_O-N were calculated by dividing the cumulative yearly CO_2_-C and N_2_O-N emissions by the annual forage yield of cash crops. Cover crop yield was not included in the calculation because they were chemically terminated and not harvested as forage.

### Statistical analysis

All GHG, soil, and water data were analyzed separately for cash crops (forage corn and sorghum) and crop phase (cover crop and cash crop) using the Mixed procedure in SAS version 9.4 (SAS Institute Inc., Cary, NC, USA). An unstructured covariance was used with Kenward–Rogers adjustment for the degrees of freedom (ddfm = kr). Crop yield, C-input from cover crops, yield-scaled CO_2_-C, and N_2_O-N, GHG_net_, and GHGI data were analyzed for cover crop-forage corn and cover crop-forage sorghum rotations considering treatment and years as fixed factors and rotation × year as a repeated term. The relationship between soil variables and GHG emissions was analyzed using simple regression analysis in SigmaPlot (V.15 Systat Software Inc., UK). Pearson’s correlation coefficient was determined using the PROC CORR procedure in SAS. All the data were tested for normality of residuals and equality of variance. The non-normal data were log-transformed, and the back-transformed means were reported. The means were separated using Fisher’s protected LSD at *p* ≤ 0.05 unless otherwise stated.

### Statement for standard protocol

All methods were carried out in accordance with relevant guidelines and regulations of the United States Department of Agriculture and New Mexico State University. The study was conducted following the standard GHG emissions and soil and plant sampling protocol.

## Supplementary Information

Supplementary Information.

## Data Availability

All data generated or analyzed during this study are included in this published article and its Supplementary Information files.
