# Supplementary material for: Net greenhouse gas balance with cover crops in semi-arid irrigated cropping systems
Source: Sci Rep. 2022 Jul 20;12:12386. doi: 10.1038/s41598-022-16719-w (PMC9300742; doi:10.1038/s41598-022-16719-w)
Supplement: Supplementary file 1 — Supplementary Information. [file 41598_2022_16719_MOESM1_ESM.docx]

**Net Greenhouse Gas Balance with Cover Crops in Semi-arid Irrigated Cropping Systems**

Pramod Acharya^a^, Rajan Ghimire^a,b,^**^*^**, Wooiklee S. Paye^b^, Amy C. Ganguli^c^, and Stephen J. DelGrosso^d^

^a^New Mexico State University, Department of Plant and Environmental Science, Las Cruces, NM 88003, USA

^b^New Mexico State University, Agricultural Science Center, Clovis, NM 88101, USA

^c^New Mexico State University, Department of Animal and Range Sciences, Las Cruces, NM 88003, USA

^d^Soil Management and Sugar Beet Research Unit, USDA-ARS, Fort Collins, CO 80526, USA

**^*^Correspondence:** [rghimire@nmsu.edu](mailto:rghimire@nmsu.edu); 2346 State Road 288, Clovis, NM 88101, USA

**SUPPLEMENTARY INFORMATION**

Number of Pages: 7

Number of Tables: 5

Number of Figures: 2

**TABLE OF CONTENTS**

| **List of Tables** | | |
| --- | --- | --- |
| **Supplementary Table S1** | Seeding rate for winter cover crop species used in each mixture in 2018/19 and 2019/20 | **2** |
| **Supplementary Table S2** | Analysis of Variance (ANOVA) showing treatment, year, and their interaction effects for average CO_2_-C and N_2_O-N emissions under different crops and phases | **3** |
| **Supplementary Table S3** | Analysis of Variance (ANOVA) showing treatment, year, and their interaction effects for average soil organic C mineralization (SOC_min_) and inorganic N at 0–0.10 m under different crops and phases | **3** |
| **Supplementary Table S4** | Analysis of Variance (ANOVA) showing treatment, year, and their interaction effects for Crop yield, carbon input, greenhouse gas balance (GHG_net_), yield-scaled CO_2_-C and N_2_O-N emissions, greenhouse gas intensity (GHGI), and cumulative GHG emissions under different crop rotations | **4** |
| **Supplementary Table S5** | Pearson's correlation coefficient between greenhouse gas emission and hydrothermal conditions (n = 212) under diverse cover crop mixture in a forage cropping system (2018–2020) | **4** |
| **List of Figures** | | |
| **Supplementary Figure S1** | Monthly average of weather data (precipitation, maximum and minimum temperatures) recorded at New Mexico State University Agricultural Science Center at Clovis Weather station and irrigation provided during the study period (2018–2020) | **5** |
| **Supplementary Figure S2** | A simple linear regression between CO_2_-C emission and environmental factors | **6** |

**Supplementary Table S1**. Seeding rate of each winter cover crop species used in each mixture in 2018/19 and 2019/20.

|  | Cover Crop Mixes* and Seed rate | | |
| --- | --- | --- | --- |
| Cover Crop Species | GBL | GB | GL |
|  | ---------------------------------kg ha^-1^---------------- | | |
| Berseem clover (var. Frosty) | 2.2 | - | 4.5 |
| Austrian winter pea | 4.5 | - | 9 |
| Annual ryegrass (var. Gulf) | 9 | 13 | 13 |
| Winter triticale (var., Forerunner) | 18 | 18 | 18 |
| Turnip | 2.2 | 2.2 | - |
| Daikon radish (var., Fracking) | 2.2 | 4.5 | - |

* (GBL)= grasses + brassicas + legumes, (GB)= grasses + brassicas, (GL)= grasses + legumes

**Supplementary Table S2**. Analysis of Variance (ANOVA) showing treatment, year, and their interaction effects for average CO_2_-C and N_2_O-N emissions under different crops and phases

| Analysis of variance (ANOVA) | CO_2_-C emission | | | | N_2_O-N emission | | | | |
| --- | --- | --- | --- | --- | --- | --- | --- | --- | --- |
|  | Forage corn | | Forage sorghum | | Forage corn | | Forage sorghum | | |
|  | Cover crop phase | Cash crop  phase | Cover crop phase | Cash crop  phase | Cover crop phase | Cash crop  phase | Cover crop phase | Cash crop  phase |  |
| Treatment (T) | **<0.0001** ^β^ | 0.277 | **<0.0001** | 0.793 | 0.599 | **0.049** | 0.298 | 0.062 |  |
| Year (Y) | **0.002** | 0.101 | **0.003** | 0.711 | **<0.0001** | **0.016** | 0.435 | **0.011** |  |
| T × Y | 0.470 | 0.403 | 0.146 | 0.695 | 0.702 | 0.149 | 0.319 | 0.141 |  |

^β^ *P*-values. Values in bold represent significantly different at *P* ≤ 0.05, LSD test.

**Supplementary Table S3**. Analysis of Variance (ANOVA) showing treatment, year, and their interaction effects for average soil organic C mineralization (SOC_min_) and inorganic N at 0–0.10 m under different crops and phases

| Analysis of variance (ANOVA) | SOC_min_ | | | | Inorganic N | | | | |
| --- | --- | --- | --- | --- | --- | --- | --- | --- | --- |
|  | Forage corn | | Forage sorghum | | Forage corn | | Forage sorghum | | |
|  | Cover crop termination | Cash crop  harvest | Cover crop termination | Cash crop  harvest | Cover crop termination | Cash crop  harvest | Cover crop termination | Cash crop  harvest |  |
| Treatment (T) | **0.013** ^β^ | 0.280 | **0.004** | 0.101 | **0.0001** | 0.708 | **0.019** | 0.581 |  |
| Year (Y) | **0.023** | 0.187 | 0.696 | 0.187 | **0.0007** | **<0.0001** | **0.002** | **<0.0001** |  |
| T × Y | 0.368 | 0.054 | 0.401 | 0.561 | 0.191 | 0.926 | 0.070 | 0.178 |  |

^β^ *P*-values. Values in bold represent significantly different at *P* ≤ 0.05, LSD test.

**Supplementary Table S4**. Analysis of Variance (ANOVA) showing treatment, year, and their interaction effects for crop yield, carbon input, greenhouse gas balance (GHG_net_), yield-scaled CO_2_-C and N_2_O-N emissions, greenhouse gas intensity (GHGI), and cumulative GHG emissions under different crop rotations

| Analysis of variance (ANOVA) | Corn yield | Sorghum yield | Cover crops aboveground C input | Yield-scaled CO_2_-C | Yield-scaled  N_2_O-N | GHG_net_ | GHGI | Cumulative CO_2_-C emissions | Cumulative N_2_O-N emissions |
| --- | --- | --- | --- | --- | --- | --- | --- | --- | --- |
| Cover crop-forage corn | | | | | | | | | |
| Treatment (T) | 0.079 ^β^ | - | 0.139 | 0.474 | 0.521 | 0.520 | 0.111 | **0.033** | 0.564 |
| Year (Y) | **0.023** | - | **0.001** | 0.211 | **<0.0001** | 0.280 | 0.859 | 0.931 | **<0.0001** |
| T × Y | 0.984 | - | **0.039** | 0.521 | 0.919 | 0.334 | 0.500 | 0.356 | 0.829 |
| Cover crop-forage sorghum | | | | | | | | | |
| Treatment (T) | - | **0.025** | 0.398 | 0.108 | 0.270 | 0.548 | 0.574 | 0.086 | 0.305 |
| Year (Y) | - | 0.568 | 0.177 | 0.555 | **0.002** | 0.086 | 0.092 | 0.470 | **0.003** |
| T × Y | - | 0.211 | 0.906 | 0.629 | 0.151 | 0.811 | 0.938 | 0.413 | 0.218 |

^β^ *P*-values. Values in bold represent significantly different at *P* ≤ 0.05, LSD test.

**Supplementary Table S5.** Pearson's correlation coefficient between greenhouse gas emission and hydrothermal conditions (n = 212) under diverse cover crop mixture in a forage cropping system (2018–2020)

| Variable | Soil temperature | Air temperature | Water-filled pore space | N_2_O-N |
| --- | --- | --- | --- | --- |
| CO_2_-C | 0.27 (<0.0001) ^†^ | 0.18 (0.011) | -0.08 (0.253) | 0.08 (0.227) |
| N_2_O-N | 0.35 (<0.0001) | 0.28 (<0.0001) | 0.31 (<0.0001) | - |

^†^ Pearson's correlation coefficient (r) followed by values in parentheses represent the *P*-value at significance level (*P* ≤ 0.05).

**Supplementary Figure S1**: Monthly average of weather data (precipitation, maximum and minimum temperatures) recorded at New Mexico State University Agricultural Science Center at Clovis Weather station and irrigation provided during the study period (2018–2020).

**Supplementary Figure S2**: Simple linear regression between CO_2_-C emission and environmental factors: A) with water-filled pore space at 0–0.1 m, B) with soil temperature at 0–0.1 m, and C) with air temperature (n=212). NCC–no cover crops control; GBL–grasses, brassicas, and legumes mixture; GB–grasses and brassicas mixture; GL–grasses and legumes mixture.
